# Supplementary material for: Spatiotemporal stop-and-go dynamics of the mitochondrial TOM core complex correlates with channel activity
Source: Commun Biol. 2022 May 17;5:471. doi: 10.1038/s42003-022-03419-4 (PMC9114391; doi:10.1038/s42003-022-03419-4)
Supplement: Supplementary file 2 — Supplementary Information [file 42003_2022_3419_MOESM2_ESM.pdf]

Supplementary Materials

Spatiotemporal *stop-and-go* dynamics of the mitochondrial TOM core complex correlates with channel activity

Shuo Wang, Lukas Findeisen, Sebastian Leptihn, Mark I. Wallace, Marcel Hörning*, Stephan Nussberger*

*Corresponding author. Email: Stephan.Nussberger@bio.uni-stuttgart.de, Marcel.Hoerning@bio.uni-stuttgart.de

This PDF file includes:

Figs. S1 to S10

Movie captions S1 to S8

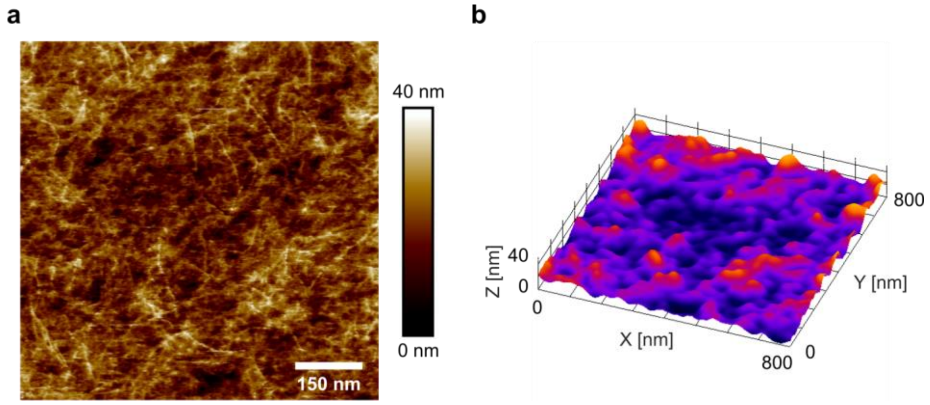

Fig. S1: AFM image of an agarose hydrogel used for TIRF imaging of supported DIB membranes. a Representative AFM image of non-modified agarose spin-coated onto a glass coverslip. **b** The surface representation of the AFM image shown in **a** shows height differences of ~ 40 nm.

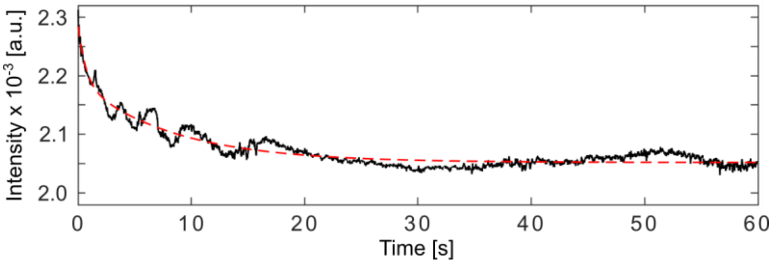

Fig. S2: Time-course of total image intensity for compensating fluorescence bleaching. Black line, average total intensity of a typical TIRF image series (512 x 512 pixels) as a function of time; red dashed line, double exponential decay used for bleach correction. The data correspond to the whole image series shown in Movie S1 and Fig.2a. a.u., arbitrary unit.

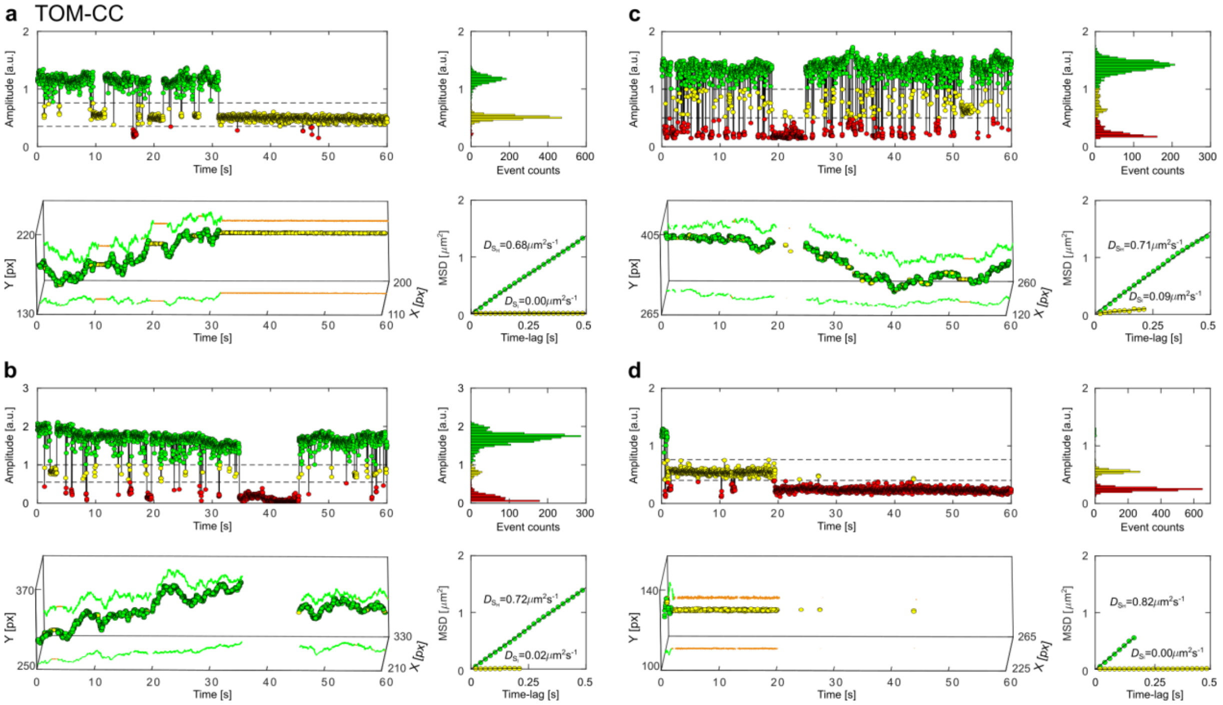

Fig. S3: Lateral mobility correlates with the open-closed channel activity of TOM-CC. **a**, **b**, **c** and **d** Representative fluorescent amplitude traces of individual TOM-CC channels recorded in non-modified agarose-supported DIB membranes. The amplitude recordings and trajectories indicate that the open-closed channel activity of TOM-CC correlates with the lateral membrane mobility of the complex. The fluorescent amplitude traces (upper left) and corresponding amplitude histograms (upper right) show three distinct ion permeation states (S_H, green; S_I, yellow; S_L, red). The trajectories (bottom left) display two mobility states, moving (green) and non-moving (yellow). The mean square displacement (MSD, bottom right) increases linearly with time when TOM-CC is in S_H state. The MSD does not change with time for TOM in the S_I state. Due to limited resolution, trajectory points and MSD-plots are not shown for TOM-CC in the low permeation state S_L. Pixel size [px], 0.16 µm. a.u., arbitrary unit.

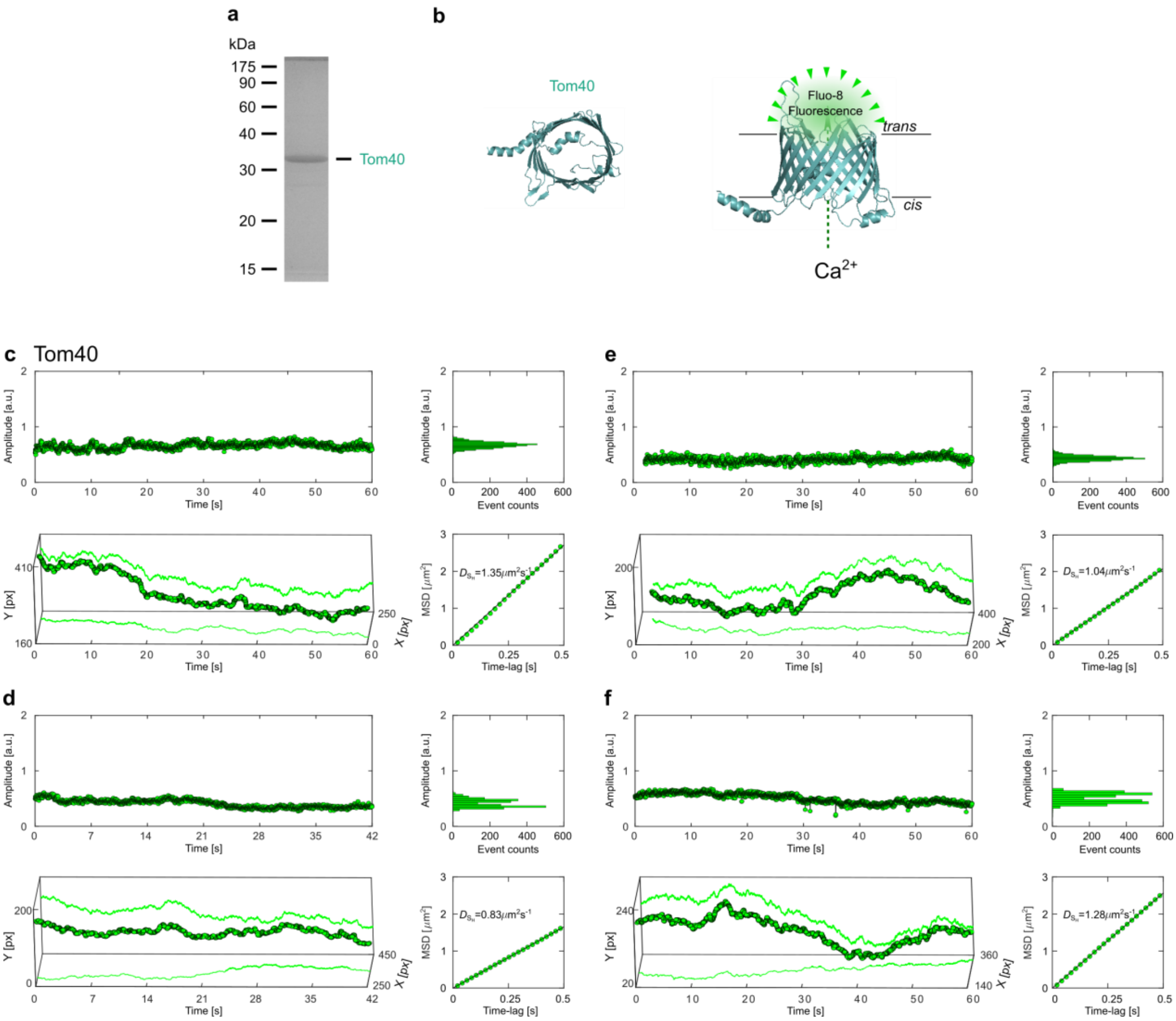

Fig. S4: Lateral mobility does not influence the open-closed channel activity of isolated Tom40. a SDS-PAGE of Tom40. The Tom40 subunit of TOM-CC was isolated from *N. crassa* mitochondria. **b** Atomic model of Tom40 (EMDB, EMD-3761; (Bausewein et al., 2020, 2017)). Left, view from the cytosolic side; right, side view. Ca^{2+} flux through the β-barrel pore is measured by electrode-free optical single channel recording using Fluo-8 as Ca^{2+}-sensitive dye, as described in Fig. 1. **c**, **d**, **e** and **f** Representative fluorescent amplitude traces of individual Tom40 channels (n = 20) recorded in agarose-supported DIB membranes. Tom40 displays only one ion permeation state and no *stop-and-go* dynamics. Pixel size [px], 0.16 µm; a.u., arbitrary unit.

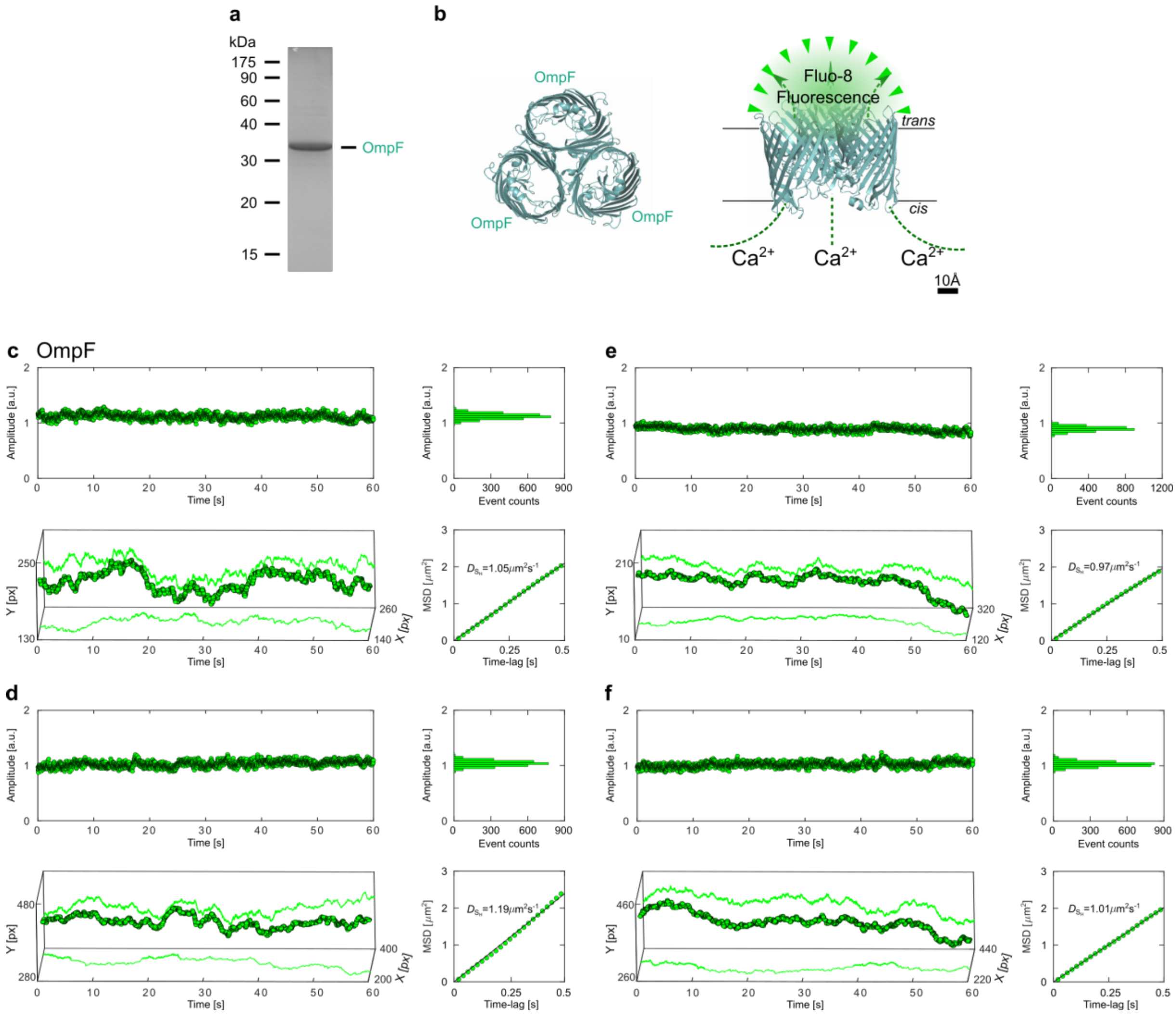

Fig. S5: Moving OmpF molecules show constant channel activity over time. a SDS-PAGE of OmpF. The protein was purified from *E. coli* membranes by differential SDS extraction at 50°C and 37°C, respectively. **b** Atomic model of OmpF (PDB, 1OPF). Left, view from the extracellular side; right, side view. Ca^{2+} flux through the three β-barrel pores is measured by electrode-free optical single channel recording using Fluo-8 as Ca^{2+}-sensitive dye, as described in Fig. 1. **c**, **d**, **e** and **f** Representative fluorescent amplitude traces of individual OmpF channels (n = 42) recorded in agarose-supported DIB membranes. OmpF displays only one ion permeation state, despite the fact that three channels are indicated in the atomic structure. Pixel size [px], 0.16 μm; a.u., arbitrary unit.

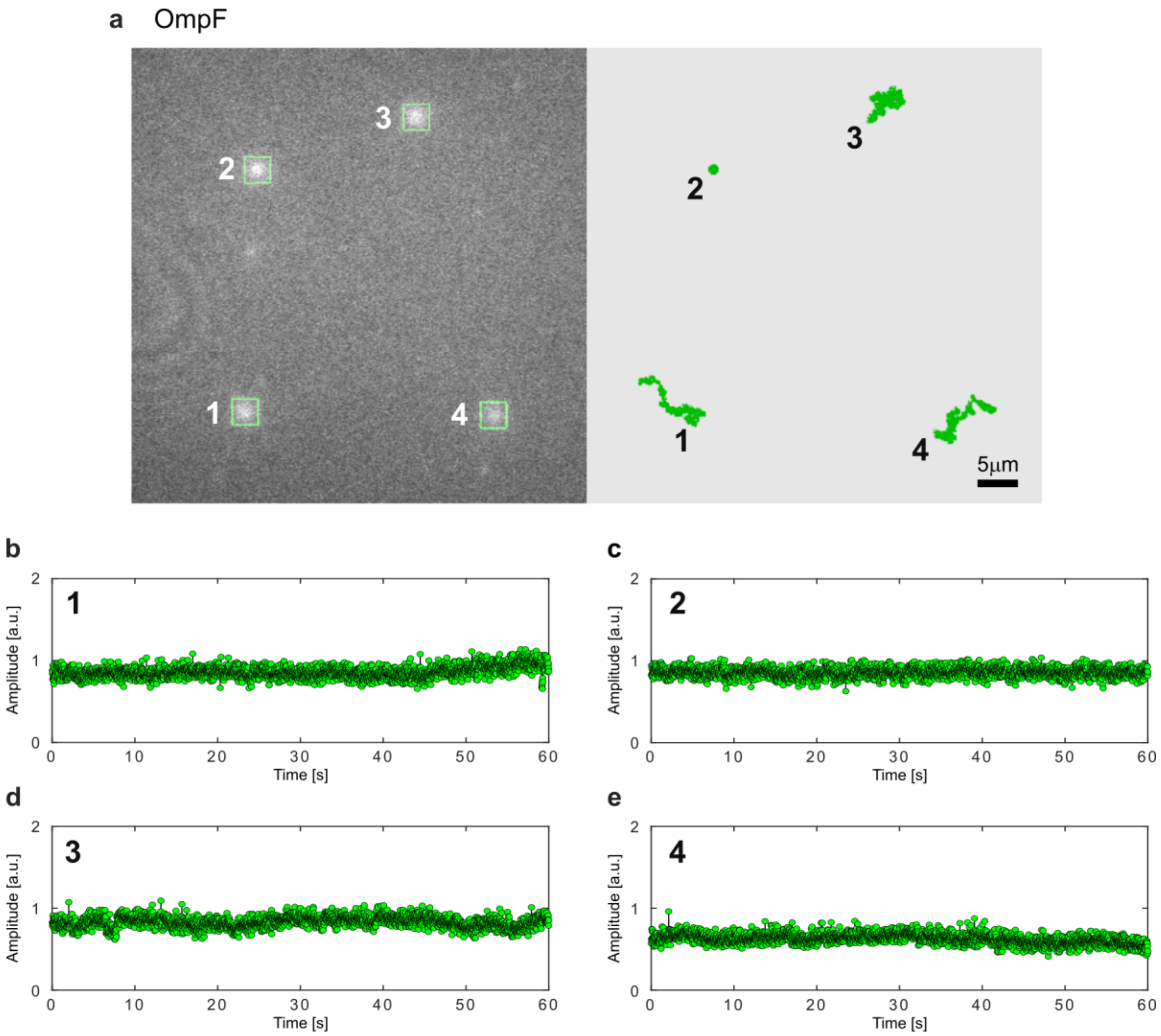

Fig. S6: Moving and non-moving OmpF molecules show similar channel activity. **a** Representative TIRF image (left) and trajectories (right) of individual OmpF molecules in a non-modified agarose supported DIB membrane. The spots show a largely constant intensity over time. The TIRF image represents a raw image that has not been corrected by fluorescence bleaching or a filter algorithm, and the individual spots are labeled 1 - 4. **b**, **c**, **d** and **e** Fluorescent amplitude traces of OmpF channels 1 - 4 shown in **a**. The amplitude recordings show that the channel activity of OmpF is similar for the moving (molecules 1, 3 and 4) and non-moving (molecule 2) OmpF molecules. A total of n = 125 moving and n = 21 permanently trapped molecules were analyzed. Original fluorescence intensities were recorded at a pixel size of 0.16 µm and at a frame rate of 47.51 s^{-1}. a.u., arbitrary unit.

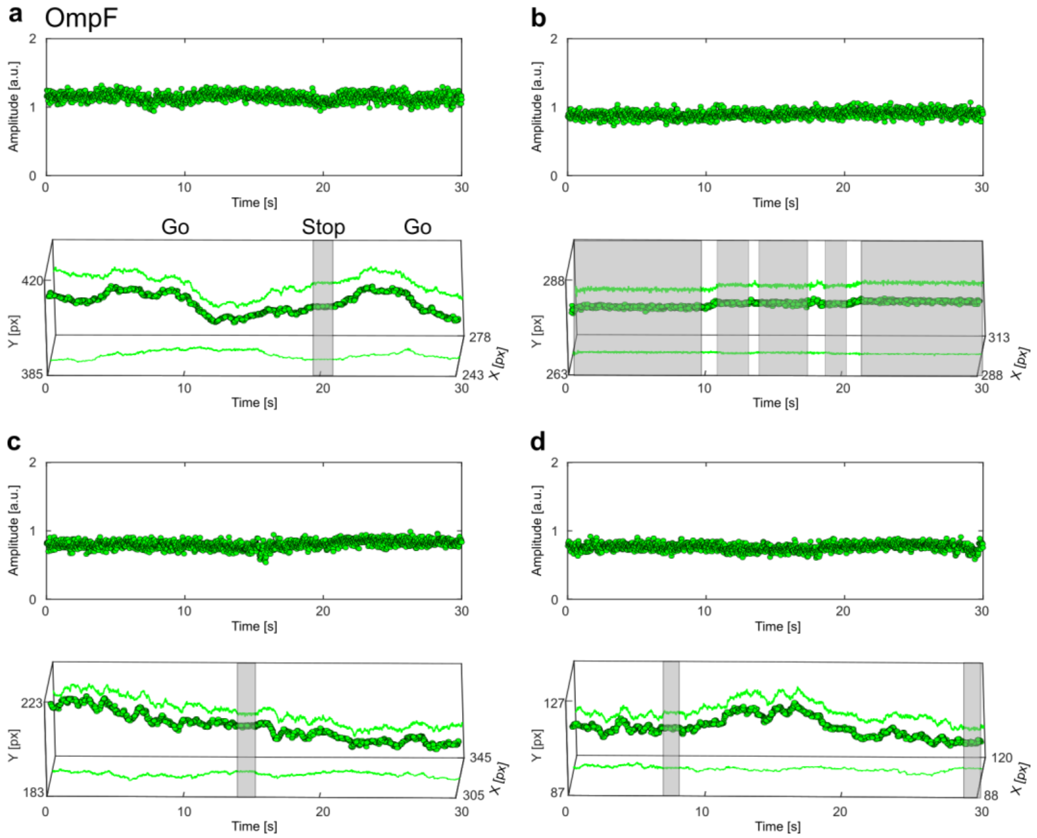

Fig. S7: ***Stop-and-go*** **movement does not influence OmpF channel activity.** **a**, **b**, **c** and **d** Representative fluorescent amplitude traces and corresponding trajectories of transiently trapped OmpF channels (*n* = 25) recorded in agarose-supported DIB membranes. OmpF shows only a single intensity level and thus only one state of ion permeation, regardless of whether it is in motion or trapped. The trajectory segments corresponding to the time periods of trapped, non-moving molecules are marked in grey. Pixel size [px], 0.16 µm; a.u., arbitrary unit.

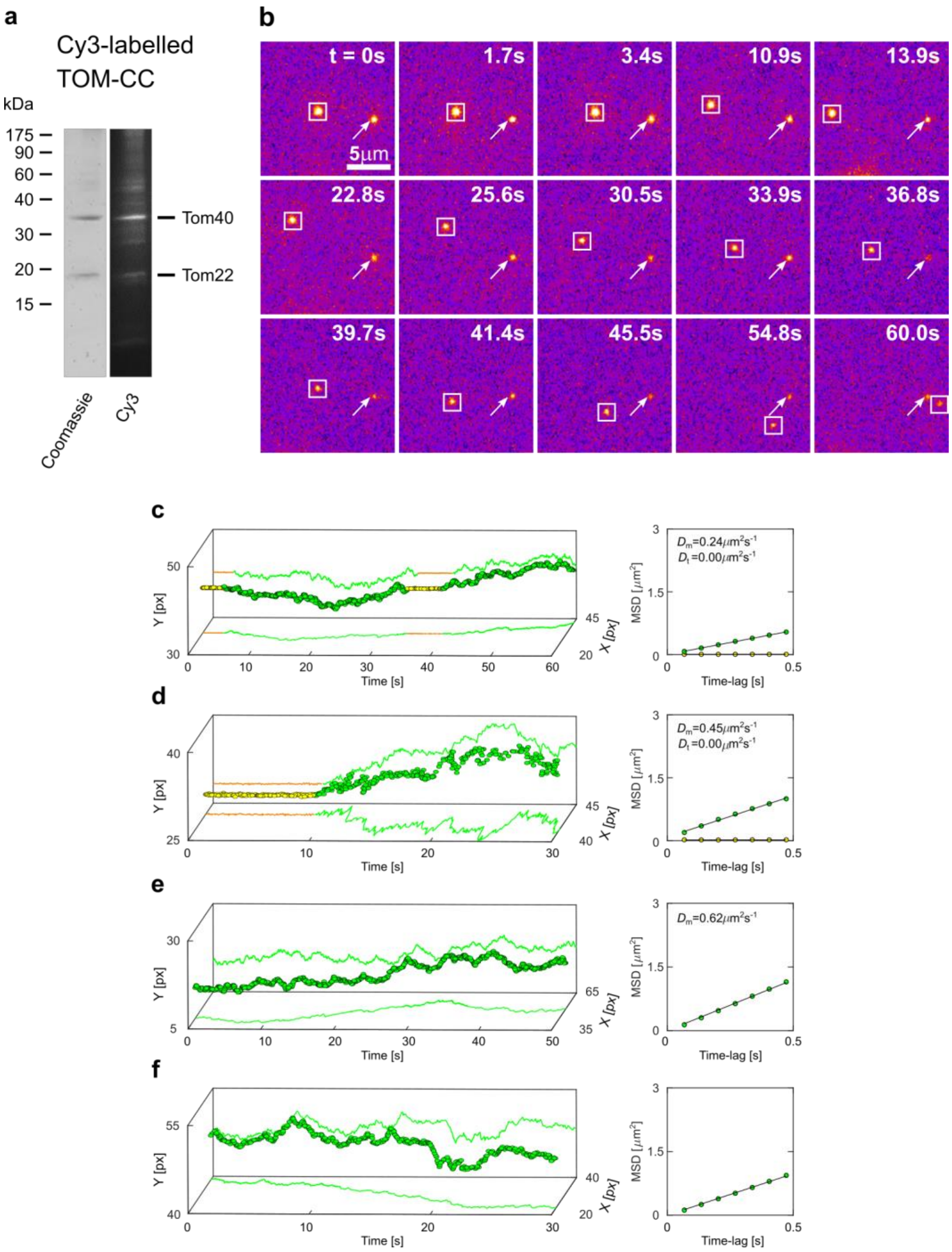

Fig. S8: Tracking fluorescent-labeled TOM-CC in non-modified agarose supported DIB membranes. a SDS-PAGE of Cy3-labeled TOM-CC analyzed by Coomassie Blue staining and Cy3 fluorescence, respectively. **b** Representative TIRF microscopy images of a DIB membrane with two Cy3-labeled TOM-CC molecules (n = 15) taken from a time series of 60 s (Movie S4). The square-marked spot displays lateral motion, interrupted by a transient arrest between t = 0 s and t = 3.3 s, and between t = 33.9 s and t = 39.4 s. The arrow-marked spot corresponds to a non-moving TOM-CC. **c** Trajectory and diffusion coefficient of the squaremarked Cy3-labeled TOM-CC. **d**, **e** and **f** Trajectories and diffusion coefficients of additional Cy3-labeled TOM-CCs. The color-coding of trajectories is the same as in Fig. 3. Note that the freely moving TOM-CC molecule in Figures **b** and **c** stops at the same spatial x,y-position when it sweeps this position a second time, indicating a specific molecular trap or anchor point at this position below the membrane. Pixel size [px], 0.16 µm.

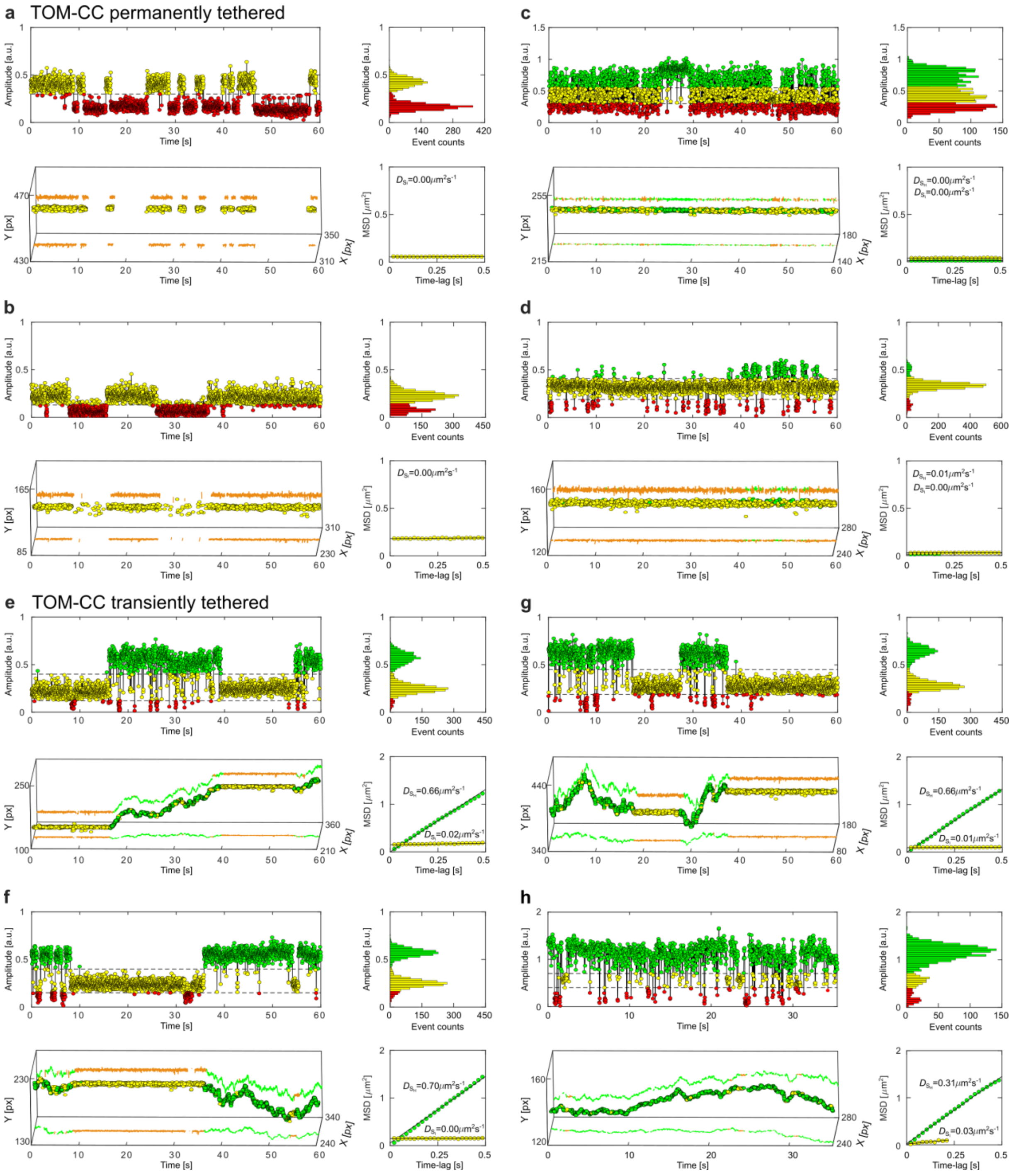

Fig. S9. Controlled immobilization of His-tagged TOM-CC triggers channel closures. Representative fluorescent amplitude traces of individual TOM-CC channels (n = 109) recorded in Ni-NTA-modified agarose-supported DIB membranes. **a**, **b**, **c** and **d** Particles are permanently trapped. **e**, **f**, **g** and **h** Particles are transiently trapped. Fluorescent amplitude traces (upper left), amplitude histograms (upper right), trajectories (bottom left) and mean square displacement plots (MSD, bottom right) are similar to those shown in Figs. 4b and 4c, respectively. The color-coding is the same as in Fig. 2. Pixel size [px], 0.16 μm; a.u., arbitrary unit.

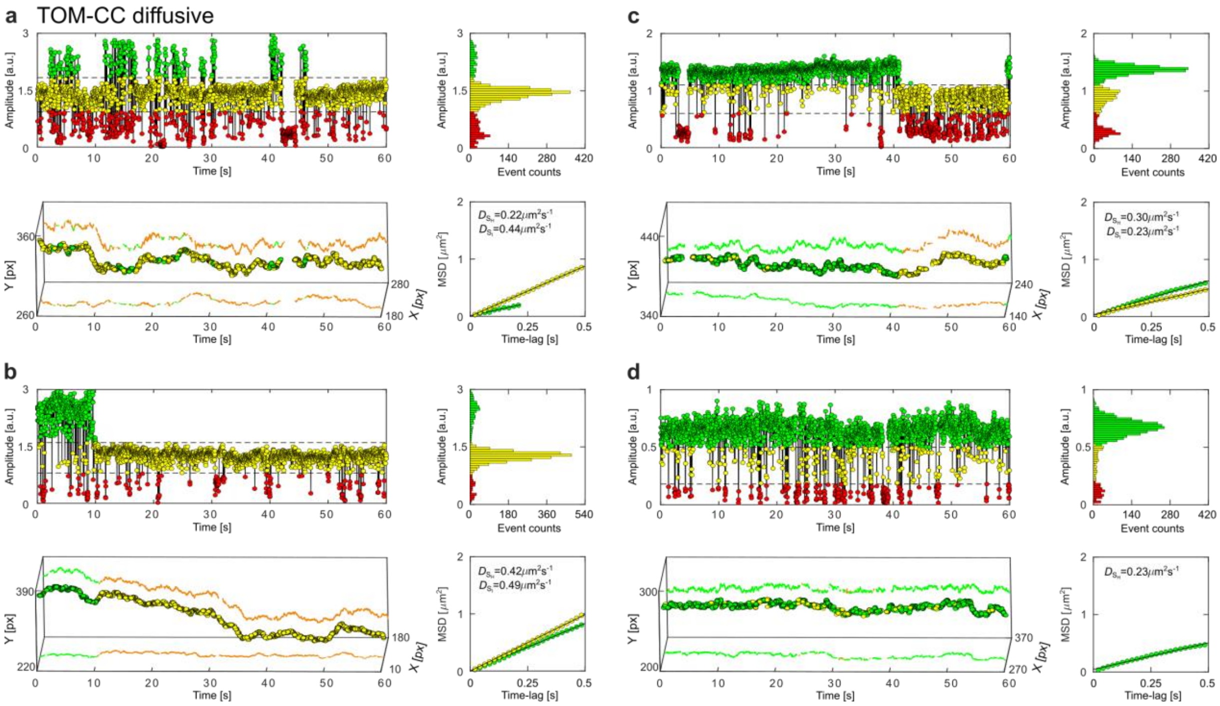

Fig. S10. Lateral mobility and open-closed channel activity of TOM-CC in Ni-NTA-modified agarose supported DIB membranes. a, **b**, **c** and **d** Fluorescent amplitude traces (upper left), amplitude histograms (upper right), trajectories (bottom left) and mean square displacement plots (MSD, bottom right) are shown. Particles (n = 14) are continuously moving and represent nontypical TOM-CCs. The color-coding is the same as in Fig. 2. a.u., arbitraty unit.

Movie S1. Imaging the channel activity of single TOM-CC molecules, related to Fig. 2a. A Ca^{2+} indicator dye (Fluo-8) is used to monitor Ca^{2+}-flux through individual TOM-CC channels in a non-modified agarose-supported DIB membrane using electrode-free optical single channel recording. TOM-CCs appear as bright spots under 488 nm TIRF-illumination. The spots show high and intermediate intensity corresponding to two conformational states S_H (green) and S_I (yellow) with two pores and one pore open, respectively. The low intensity level represents a conformation S_L (red) with both pores closed. The intensity trace of the upper right spot is shown in Movie S2 and in Fig.2b. The probabilities for TOM-CC being in states S_H, S_I and S_L are presented in Fig.5. Raw image data are shown, i.e. the video sequence was neither corrected by fluorescence bleaching nor by a filter algorithm; individual spots are marked according to their conformational states.

Movie S2. Time evolution of TOM-CC channel activity, related to Figs. 2b – 2c. Fitting the fluorescence intensity profiles of a single TOM-CC to two-dimensional Gaussian functions (right). Red, yellow and green intensity profiles represent TOM-CC in S_L, S_I and S_H demonstrating Tom40 channels, which are fully closed, one, and two channels open, respectively. Original fluorescence intensities (left) were recorded at a pixel size of 0.16 μm and at a frame rate of 47.51 s^{-1}. No bleach correction and filter algorithm were applied.

Movie S3. Correlation between stop-and-go dynamics and open-closed channel activity of single TOM-CC molecules, related to Fig. 3. TIRF image recording (left) and trajectories (right) of individual TOM-CC molecules in a non-modified agarose supported DIB membrane. The square-marked spots display lateral motion (Go) interrupted by transient arrest (Stop). The red, yellow and green color coding corresponds to TOM-CC molecules, which are fully closed (S_L), one (S_I) and two (S_H) channels open, respectively. Moving TOM-CC molecules in S_H switch to S_I or S_L when they stop in the DIB membrane. Raw image data are shown, i.e. the video sequence was neither corrected by fluorescence bleaching nor by a filter algorithm; individual spots are marked according to their conformational states (left). The trajectories of moving TOM-CC molecules are colored in green; the trajectories of trapped TOM-CC molecules in S_I are colored in yellow; the trajectories of trapped TOM-CC molecules in S_L are not shown because weak intensity profiles do not allow accurate determination of the position of TOM-CC in the membrane plane.

Movie S4. Stop-and-go movement of fluorescently labeled TOM-CC, related to Fig. S4. TIRF image recording (left) and trajectories (right) of Cy3-labeled TOM-CC molecules in a non-modified agarose supported DIB membrane. The square-marked spots display lateral motion (Go, green), interrupted by a transient arrest (Stop, yellow). Note that the freely moving TOMCC molecule stops at the same spatial x,y-position (yellow cross) when it crosses the same position a second time, indicating a specific molecular trap or anchor point at this position below the membrane. Raw image data are shown. Grey scales of individual images are transformed into pseudo color images to better display movement of fluorescently labelled TOM-CC molecules. No bleach correction and filter algorithm were applied.

Movie S5. Lateral movement and channel activity of single Tom40 molecules, related to Figs. 2e – 2f, Fig. 3e and Fig.S2. TIRF image recording (left) and trajectories (right) of single isolated Tom40 molecules in a non-modified agarose supported DIB membrane. A Ca^{2+} indicator dye (Fluo-8) is used to monitor Ca^{2+}-flux through single β-barrel Tom40 channels using electrode-free optical single channel recording. Tom40 appears as bright spots

under 488 nm TIRF illumination. Tom40 displays only one ion permeation and lateral membrane mobility state. The non-highlighted moving spot with low fluorescence intensity could be a residual moving class II TOM-CC with non-functional Tom22 as described in Fig. 5, or alternatively a non-functional Tom40. Raw image data are shown and individual spots are marked. No bleach correction and filter algorithm were applied.

Movie S6. Lateral movement and channel activity of single OmpF molecules, related to Figs. 2e – 2f, and Fig. S3. TIRF image recording (left) and trajectories (right) of single OmpF molecules in a non-modified agarose supported DIB membrane. A Ca^{2+} indicator dye (Fluo-8) is used to monitor Ca^{2+}-flux through three-pore β-barrel OmpF channels using electrode-free optical single channel recording. OmpF appears as bright spots under 488 nm TIRF illumination. The channel activity of OmpF does not correlate with the lateral mobility of the protein. OmpF displays only one ion permeation and lateral membrane mobility state. Raw image data are shown and individual spots are marked. No bleach correction and filter algorithm were applied.

Movie S7. Single channel activity of transiently and permanently trapped TOM-CC molecules, related to Figs. 3, 4 and 5. TIRF image recording (left) and trajectories (right) of single His-tagged TOM-CC molecules in non-modified (top) and Ni-NTA-modified (bottom) agarose supported DIB membranes. The spots show high and intermediate intensity corresponding to two conformational states S_H (green) and S_I (yellow) with two pores and one pore open, respectively. The low intensity level represents a conformation S_L (red) with both pores closed. Diffusive TOM-CC molecules are only in S_H state. Non-diffusive molecules are either in S_I or S_L. The permanently tethered fraction of TOM-CC in Ni-NTA-modified agarose is significantly larger compared to the fraction in non-modified agarose over time. Raw image data are shown, i.e. no bleach correction and filter algorithm were applied; individual spots are marked according to their conformational states; trajectories of trapped TOM-CC molecules in S_L are not shown because weak intensity profiles do not allow accurate determination of the position of TOM-CC in the membrane plane. The Ni-NTA agarose was custom synthesized. The Ni-ion of the Ni-NTA reduces the signal-to-noise ratio. Original fluorescence intensities were recorded at a pixel size of 0.16 μm and at a frame rate of 47.51 s^{-1}.

Movie S8. Lateral movement and channel activity of His-tagged TOM-CC molecules in Ni-NTA-modified agarose supported DIB membranes in the presence of imidazole. TIRF image recording (left) and trajectories (right) of single TOM-CC molecules. The spots show constant intensity corresponding to conformational state S_H and no permanent binding of His-tagged TOM-CC to the Ni-NTA-modified agarose due to the presence of imidazole. Raw image data are shown, i.e. no bleach correction and filter algorithm were applied. The Ni-NTA agarose was custom synthesized. The blue color of the Ni-NTA reduces the signal-to-noise ratio. Original fluorescence intensities were recorded at a pixel size of 0.16 μm and at a frame rate of 47.51 s^{-1}.
