# Supplementary material for: Spatiotemporal stop-and-go dynamics of the mitochondrial TOM core complex correlates with channel activity
Source: Commun Biol. 2022 May 17;5:471. doi: 10.1038/s42003-022-03419-4 (PMC9114391; doi:10.1038/s42003-022-03419-4)
Supplement: Supplementary file 3 — Description of Additional Supplementary Files [file 42003_2022_3419_MOESM3_ESM.pdf]

Description of Additional Supplementary Files

File name: Movie S1
Description: Imaging the channel activity of single TOM-CC molecules, related to Fig. 2a. A Ca2+ indicator dye (Fluo-8) is used to monitor Ca2+ -flux through individual TOM-CC channels in a non-modified agarose-supported DIB membrane using electrode-free optical single channel recording. TOM-CCs appear as bright spots under 488 nm TIRF-illumination. The spots show high and intermediate intensity corresponding to two conformational states SH (green) and SI (yellow) with two pores and one pore open, respectively. The low intensity level represents a conformation SL (red) with both pores closed. The intensity trace of the upper right spot is shown in Movie S2 and in Fig.2b. The probabilities for TOM-CC being in states SH, SI and SL are presented in Fig.5. Raw image data are shown, i.e. the video sequence was neither corrected by fluorescence bleaching nor by a filter algorithm; individual spots are marked according to their conformational states.

File name: Movie S2
Description: Time evolution of TOM-CC channel activity, related to Figs. 2b – 2c. Fitting the fluorescence intensity profiles of a single TOM-CC to two-dimensional Gaussian functions (right). Red, yellow and green intensity profiles represent TOM-CC in SL, SI and SH demonstrating Tom40 channels, which are fully closed, one, and two channels open, respectively. Original fluorescence intensities (left) were recorded at a pixel size of 0.16 μm and at a frame rate of 47.51 s-1 . No bleach correction and filter algorithm were applied.

File name: Movie S3
Description: Correlation between stop-and-go dynamics and open-closed channel activity of single TOM-CC molecules, related to Fig. 3. TIRF image recording (left) and trajectories (right) of individual TOM-CC molecules in a non-modified agarose supported DIB membrane. The square-marked spots display lateral motion (Go) interrupted by transient arrest (Stop). The red, yellow and green color coding corresponds to TOM-CC molecules, which are fully closed (SL), one (SI) and two (SH) channels open, respectively. Moving TOMCC molecules in SH switch to SI or SL when they stop in the DIB membrane. Raw image data are shown, i.e. the video sequence was neither corrected by fluorescence bleaching nor by a filter algorithm; individual spots are marked according to their conformational states (left). The trajectories of moving TOM-CC molecules are colored in green; the trajectories of trapped TOM-CC molecules in SI are colored in yellow; the trajectories of trapped TOM-CC molecules in SL are not shown because weak intensity profiles do not allow accurate determination of the position of TOM-CC in the membrane plane.

File name: Movie S4
Description: Stop-and-go movement of fluorescently labeled TOM-CC, related to Fig. S4. TIRF image recording (left) and trajectories (right) of Cy3-labeled TOM-CC molecules in a non-modified agarose supported DIB membrane. The square-marked spots display lateral motion (Go, green), interrupted by a transient arrest (Stop, yellow). Note that the freely moving TOMCC molecule stops at the same spatial x,y-position (yellow cross) when it crosses the same position a second time, indicating a specific molecular trap or anchor point at this position below the membrane. Raw image data are shown. Grey scales of individual images are transformed into pseudo color images to better display movement of fluorescently labelled TOM-CC molecules. No bleach correction and filter algorithm were applied.

File name: Movie S5

Description: Lateral movement and channel activity of single Tom40 molecules, related to Figs. 2e – 2f, Fig. 3e and Fig.S2. TIRF image recording (left) and trajectories (right) of single isolated Tom40 molecules in a non-modified agarose supported DIB membrane. A Ca2+ indicator dye (Fluo-8) is used to monitor Ca2+ -flux through single β-barrel Tom40 channels using electrode-free optical single channel recording. Tom40 appears as bright spots 13 under 488 nm TIRF illumination. Tom40 displays only one ion permeation and lateral membrane mobility state. The non-highlighted moving spot with low fluorescence intensity could be a residual moving class II TOM-CC with non-functional Tom22 as described in Fig. 5, or alternatively a non-functional Tom40. Raw image data are shown and individual spots are marked. No bleach correction and filter algorithm were applied.

File name: Movie S6

Description: Lateral movement and channel activity of single OmpF molecules, related to Figs. 2e – 2f, and Fig. S3. TIRF image recording (left) and trajectories (right) of single OmpF molecules in a non-modified agarose supported DIB membrane. A Ca2+ indicator dye (Fluo-8) is used to monitor Ca2+ -flux through three-pore β-barrel OmpF channels using electrode-free optical single channel recording. OmpF appears as bright spots under 488 nm TIRF illumination. The channel activity of OmpF does not correlate with the lateral mobility of the protein. OmpF displays only one ion permeation and lateral membrane mobility state. Raw image data are shown and individual spots are marked. No bleach correction and filter algorithm were applied.

File name: Movie S7

Description: Single channel activity of transiently and permanently trapped TOM-CC molecules, related to Figs. 3, 4 and 5. TIRF image recording (left) and trajectories (right) of single His-tagged TOM-CC molecules in non-modified (top) and Ni-NTA-modified (bottom) agarose supported DIB membranes. The spots show high and intermediate intensity corresponding to two conformational states SH (green) and SI (yellow) with two pores and one pore open, respectively. The low intensity level represents a conformation SL (red) with both pores closed. Diffusive TOM-CC molecules are only in SH state. Non-diffusive molecules are either in SI or SL. The permanently tethered fraction of TOM-CC in Ni-NTAmodified agarose is significantly larger compared to the fraction in non-modified agarose over time. Raw image data are shown, i.e. no bleach correction and filter algorithm were applied; individual spots are marked according to their conformational states; trajectories of trapped TOM-CC molecules in SL are not shown because weak intensity profiles do not allow accurate determination of the position of TOM-CC in the membrane plane. The Ni-NTA agarose was custom synthesized. The Ni-ion of the Ni-NTA reduces the signal-to-noise ratio. Original fluorescence intensities were recorded at a pixel size of 0.16 μm and at a frame rate of 47.51 s-1 .

File name: Movie S8

Description: Lateral movement and channel activity of His-tagged TOM-CC molecules in Ni-NTA-modified agarose supported DIB membranes in the presence of imidazole. TIRF image recording (left) and trajectories (right) of single TOM-CC molecules. The spots show constant intensity corresponding to conformational state SH and no permanent binding of His-tagged TOM-CC to the Ni-NTA-modified agarose due to the presence of imidazole. Raw image data are shown, i.e. no bleach correction and filter algorithm were applied. The Ni-NTA agarose was custom synthesized. The blue color of the Ni-NTA reduces the signalto-noise ratio. Original fluorescence intensities were recorded at a pixel size of 0.16 μm and at a frame rate of 47.51 s-1 .
