# Supplementary material for: Spatiotemporal stop-and-go dynamics of the mitochondrial TOM core complex correlates with channel activity
Source: Commun Biol. 2022 May 17;5:471. doi: 10.1038/s42003-022-03419-4 (PMC9114391; doi:10.1038/s42003-022-03419-4)
Supplement: Supplementary file 12 — Reporting Summary [file 42003_2022_3419_MOESM12_ESM.pdf]

Corresponding author(s): Stephan Nussberger, Marcel Hörning

Last updated by author(s): Apr 17, 2022

Reporting Summary

Nature Portfolio wishes to improve the reproducibility of the work that we publish. This form provides structure for consistency and transparency in reporting. For further information on Nature Portfolio policies, see our Editorial Policies and the Editorial Policy Checklist.

Statistics

For all statistical analyses, confirm that the following items are present in the figure legend, table legend, main text, or Methods section.

n/a	Confirmed	
☐	☒	The exact sample size (*n*) for each experimental group/condition, given as a discrete number and unit of measurement
☐	☒	A statement on whether measurements were taken from distinct samples or whether the same sample was measured repeatedly
☒	☐	The statistical test(s) used AND whether they are one- or two-sided<br>*Only common tests should be described solely by name; describe more complex techniques in the Methods section.*
☒	☐	A description of all covariates tested
☒	☐	A description of any assumptions or corrections, such as tests of normality and adjustment for multiple comparisons
☐	☒	A full description of the statistical parameters including central tendency (e.g. means) or other basic estimates (e.g. regression coefficient) AND variation (e.g. standard deviation) or associated estimates of uncertainty (e.g. confidence intervals)
☒	☐	For null hypothesis testing, the test statistic (e.g. *F*, *t*, *r*) with confidence intervals, effect sizes, degrees of freedom and *P* value noted<br>*Give P values as exact values whenever suitable.*
☒	☐	For Bayesian analysis, information on the choice of priors and Markov chain Monte Carlo settings
☒	☐	For hierarchical and complex designs, identification of the appropriate level for tests and full reporting of outcomes
☒	☐	Estimates of effect sizes (e.g. Cohen's *d*, Pearson's *r*), indicating how they were calculated

Our web collection on statistics for biologists contains articles on many of the points above.

Software and code

Policy information about availability of computer code

Data collection	TIRF image collection: NIS-Elements AR (Nikon, Japan, https://www.nikon.com)
Data analysis	Matlab R2020a (The Mathworks, Natick, MA, https://de.mathworks.com/downloads/)

For manuscripts utilizing custom algorithms or software that are central to the research but not yet described in published literature, software must be made available to editors and reviewers. We strongly encourage code deposition in a community repository (e.g. GitHub). See the Nature Portfolio guidelines for submitting code & software for further information.

Data

Policy information about availability of data

All manuscripts must include a data availability statement. This statement should provide the following information, where applicable:

- Accession codes, unique identifiers, or web links for publicly available datasets
- A description of any restrictions on data availability
- For clinical datasets or third party data, please ensure that the statement adheres to our policy

All data generated or analyzed during this study are included in the manuscript and supporting files. All original TIRF source data are stored at the data repository of the University of Stuttgart (DaRUS): Shuo Wang, Lukas Findeisen, Sebastian Leptihn, Mark I. Wallace, Marcel Hörning, Stephan Nussberger, 2021. Data for: Correlation of mitochondrial TOM core complex stop-and-go and open-closed channel dynamics, https://doi.org/10.18419/darus-2158, DaRUS data repository of the University of Stuttgart doi:10.18419/darus-2158.

Field-specific reporting

Please select the one below that is the best fit for your research. If you are not sure, read the appropriate sections before making your selection.

☒ Life sciences ☐ Behavioural & social sciences ☐ Ecological, evolutionary & environmental sciences

For a reference copy of the document with all sections, see nature.com/documents/nr-reporting-summary-flat.pdf

Life sciences study design

All studies must disclose on these points even when the disclosure is negative.

Sample size	Data of individual TOM-CCs in DIB membranes supported by non-modified agarose were taken from 27 independent experiments/movies (n = 64 TOM-CC molecules) and 60 s recording time, respectively (Fig. 5 and Results section).<br><br>Data of individual TOM-CCs in DIB membranes supported by Ni-NTA-modified agarose were taken from 33 independent experiments/movies (n = 123 TOM-CC molecules) and 60 s recording time, respectively (Fig. 5 and Results section).<br><br>Data of individual OmpF channels in DIB membranes supported by non-modified agarose were taken from 66 independent experiments/ movies (n = 171 OmpF molecules) and 60 s recording time, respectively (Figs. 2e, 2e, S5 – S7 and Results section).<br><br>Data of individual Cy3 labeled TOM-CCs in DIB membranes supported by non-modified agarose were taken from 8 independent experiments/ movies (n = 15 TOM-CC molecules) and 60 s recording time, respectively (Figs. S8b – S8f and Results section).<br><br>Data of individual Tom40 channels in DIB membranes supported by non-modified agarose were taken from 8 independent experiments/ movies (n = 20 Tom40 molecules) and 60 s recording time, respectively (Figs. S4b – S4f and Results section).<br><br>Data of individual TOM-CCs in DIB membranes supported by Ni-NTA-modified agarose in the presence of imidazole were taken from 4 independent experiments/movies (n = 31 TOM-CC molecules) and 60 s recording time, respectively.
Data exclusions	TIRF signals representing adjacent TOM-CC molecules that could not clearly be separated by Gaussian fitting were not evaluated.
Replication	No technical replications were recorded from the same TOM-CC, Tom40 and OmpF molecules.
Randomization	N/A
Blinding	N/A

Reporting for specific materials, systems and methods

We require information from authors about some types of materials, experimental systems and methods used in many studies. Here, indicate whether each material, system or method listed is relevant to your study. If you are not sure if a list item applies to your research, read the appropriate section before selecting a response.

Materials & experimental systems

n/a	Involved in the study	
☒	☐	Antibodies
☐	☒	Eukaryotic cell lines
☒	☐	Palaeontology and archaeology
☒	☐	Animals and other organisms
☒	☐	Human research participants
☒	☐	Clinical data
☒	☐	Dual use research of concern

Methods

n/a	Involved in the study	
☒	☐	ChIP-seq
☒	☐	Flow cytometry
☒	☐	MRI-based neuroimaging

Eukaryotic cell lines

Policy information about cell lines

Cell line source(s)	Neurospora crassa
Authentication	T107
Mycoplasma contamination	N/A

Commonly misidentified lines<br>(See ICLAC register)	N/A
